# ^18^F-FDG-labeled red blood cell PET for blood-pool imaging: preclinical evaluation in rats

**DOI:** 10.1186/s13550-017-0266-3

**Published:** 2017-02-27

**Authors:** Yohji Matsusaka, Tadaki Nakahara, Kazuhiro Takahashi, Yu Iwabuchi, Chiyoko Nishime, Mayumi Kajimura, Masahiro Jinzaki

**Affiliations:** 1 Department of Diagnostic Radiology, Keio University School of Medicine, 35 Shinanomachi, Shinjuku-ku, Tokyo, 160-8582 Japan; 2 Central Institute for Experimental Animals, Kawasaki-ku, Kawasaki, Kanagawa 210-0821 Japan; 3 Department of Biology, Keio University School of Medicine, Hiyoshi, Kohoku-ku, Yokohama, Kanagawa 223-8522 Japan

**Keywords:** Red blood cell, ^18^F-FDG, Blood-pool imaging, Intraabdominal bleeding, PET

## Abstract

**Background:**

Red blood cells (RBCs) labeled with single-photon emitters have been clinically used for blood-pool imaging. Although some PET tracers have been introduced for blood-pool imaging, they have not yet been widely used. The present study investigated the feasibility of labeling RBCs with ^18^F-2-deoxy-2-fluoro-D-glucose (^18^F-FDG) for blood-pool imaging with PET.

RBCs isolated from venous blood of rats were washed with glucose-free phosphate-buffered saline and labeled with ^18^F-FDG. To optimize labeling efficiency, the effects of glucose deprivation time and incubation (labeling) time with ^18^F-FDG were investigated. Post-labeling stability was assessed by calculating the release fraction of radioactivity and identifying the chemical forms of ^18^F in the released and intracellular components of ^18^F-FDG-labeled RBCs incubated in plasma. Just after intravenous injection of the optimized autologous ^18^F-FDG-labeled RBCs, dynamic PET scans were performed to evaluate in vivo imaging in normal rats and intraabdominal bleeding models (temporary and persistent bleeding).

**Results:**

The optimal durations of glucose deprivation and incubation (labeling) with ^18^F-FDG were 60 and 30 min, respectively. As low as 10% of ^18^F was released as the form of ^18^F-FDG from ^18^F-FDG-labeled RBCs after a 60-min incubation. Dynamic PET images of normal rats showed strong persistence in the cardiovascular system for at least 120 min. In the intraabdominal bleeding models, ^18^F-FDG-labeled RBC PET visualized the extravascular blood clearly and revealed the dynamic changes of the extravascular radioactivity in the temporary and persistent bleeding.

**Conclusions:**

RBCs can be effectively labeled with ^18^F-FDG and used for blood-pool imaging with PET in rats.

**Electronic supplementary material:**

The online version of this article (doi:10.1186/s13550-017-0266-3) contains supplementary material, which is available to authorized users.

## Background

Nuclear medicine techniques using red blood cells (RBCs) labeled with several single-photon emitters such as ^99m^Tc have been used for blood-pool imaging (BPI). The purposes of BPI include the detection of gastrointestinal bleeding [1, 2], evaluation of cardiac function [3, 4], localization of hemangiomas [5, 6], and regional cerebral blood volume measurement [7]. RBCs would be a more suitable tracer for BPI than radionuclide-labeled plasma proteins because plasma proteins tend to leak into extravascular spaces [8]. Since Fisher et al. reported the first human study of ^99m^Tc-labeled RBCs for splenic radionuclide imaging 50 years ago [9], ^99m^Tc-labeled RBC scintigraphy has been clinically used for BPI. Furthermore, single-photon emission computed tomography/computed tomography (SPECT/CT) improves the anatomical information of ^99m^Tc-labeled RBC imaging [10].

Although positron emission tomography (PET) has better image quality than scintigraphy and SPECT, blood-pool agents for PET are limited. Carbon monoxide (CO) labeled with ^11^C or ^15^O has been applied to RBC imaging with PET [11, 12], but the short half-life of these radionuclides prevents their widespread use. Fluorine-18 seems more suitable for PET BPI in terms of the longer half-life and shorter positron range [13]. To our knowledge, there have been a few reports which demonstrated the labeling of RBCs with ^18^F-labeled compounds: ^18^F-sulfonamide derivative [14] and ^18^F-N-succinimidyl 4-^18^F-fluorobenzoate (^18^F-SFB) [13]. In these two reports, RBC PET was successful in evaluation of cardiac function or blood volume quantification, respectively.

Fluorine-18-2-deoxy-2-fluoro-D-glucose (^18^F-FDG) is the most available PET tracer in the world. Fluorine-18-FDG-labeled white blood cells (WBCs) have already been clinically used as an inflammation-seeking agent [15–18]. Due to the absence of nuclei or mitochondria, glucose is the only energy substrate for RBCs; thus, we speculated that RBCs could be also labeled with ^18^F-FDG for imaging. There has been evidence of transporting ^18^F-FDG into RBCs in an equilibrium state [19, 20]. The aims of the present study were to determine an optimal labeling procedure for ^18^F-FDG-labeled RBCs and to demonstrate the suitability of the RBC preparation for BPI using in vivo rat imaging.

## Methods

### Animals

The entire experimental protocols were approved by the Keio University Institutional Animal Care and Use Committee and performed in accordance with the Institutional Guidelines on Animal Experimentation at Keio University. Healthy male F344/Jcl rats were purchased from Clea Japan Incorporation. The rats were given standard rat chow and tap water ad libitum.

### RBC preparation for in vitro studies

After a 3-h fasting period, the rats (weight, 188.7 ± 16 g) were anesthetized with isoflurane. The inferior vena cava (IVC) was exposed through an abdominal incision. A 23-gauge needle was placed in the IVC, and heparin (1000 IU/kg body weight) was administered to prevent blood coagulation. Thirty seconds later, the blood (4 ml) was slowly withdrawn from the IVC. Blood glucose concentration was measured with a glucometer (Accu-Chek Aviva; Roche Diagnostics Co., Ltd., Japan). The blood was centrifuged at 500*g* for 10 min at 4 °C. The supernatant (plasma) was stored for subsequent post-labeling stability studies. The buffy coat, which contains most of WBCs and platelets, was removed. To eliminate extracellular glucose, the packed RBCs were washed three times in the following way: RBCs were mixed with glucose-free phosphate-buffered saline (PBS) (8 ml; Wako Pure Chemical Industries, Ltd., Japan) and centrifuged at 1000*g* for 2 min, and the supernatant was removed. After washing, the packed RBC suspension was uniformly resuspended and the hematocrit was measured with the microhematocrit technique after centrifuging at 12,000*g* for 3 min [21, 22]. Hematocrit was adjusted to 2.5–80% by diluting the packed RBCs with a calculated volume of PBS.

### Preparation of ^18^F-FDG

Fluorine-18-FDG was produced in a clinical routine setup on-site. The specific radioactivity at the time of labeling was approximately 500 GBq/μmol. The osmolarity of ^18^F-FDG solution was adjusted to prevent some RBCs from rupture because of the hyposmosis of original ^18^F-FDG solution (less than 4 mOsm/L). As previously performed in the study of ^18^F-FDG-labeled WBCs by Osman et al. [15], 11 μL of 10X PBS (2800 mOsm/L; Wako Pure Chemical Industries, Ltd., Japan) was added to 100 μL of ^18^F-FDG solution.

### Labeling efficiency analysis

We considered that labeling efficiency (LE) of ^18^F-FDG-labeled RBCs would be influenced by the following possible factors: depletion of intracellular glucose before labeling, hematocrit for labeling, and incubation (labeling) time with ^18^F-FDG.

We hypothesized that LE could be increased by incubating RBCs in glucose-free PBS at 37 °C before labeling, which can reduce intracellular glucose concentration. To investigate the effect of glucose deprivation before labeling, the prepared RBC suspension (70% hematocrit, 100 μL) was incubated at 37 °C for 0–120 min. The incubated RBC suspension was mixed with ^18^F-FDG solution (12.0 ± 1.7 MBq, 15.3 ± 0.3 μL, *n* = 4) and incubated for 30 min at 37 °C for labeling. To eliminate extracellular ^18^F-FDG, the labeled RBCs were washed three times at 4 °C with Hanks’ balanced salt solution (HBSS) (Wako Pure Chemical Industries, Ltd., Japan) containing glucose (100 mg/dL). The primary radioactivity of ^18^F-FDG and the radioactivity of the labeled RBCs were measured with a dose calibrator (Curiemeter IGC-7; Aloka, Japan). Then, LE was calculated as a percentage by dividing the radioactivity of the labeled RBCs by the primary radioactivity with decay correction.

The relationship between hematocrit for labeling and LE was analyzed. The hematocrit of the prepared RBC suspension was adjusted to 2.5–80%. After a 60-min incubation at 37 °C for reducing intracellular glucose concentration before labeling, each hematocrit suspension (100 μL) was mixed with ^18^F-FDG solution (15.0 MBq, 14.6 μL) and incubated for 30 min at 37 °C for labeling. Washing, radioactivity measurement, and LE calculation were performed following the same procedure as described above. In addition, the relationship between the added radioactivity of ^18^F-FDG (7.3–20.1 MBq) and the radioactivity of labeled RBCs was analyzed.

The effect of the labeling (incubation) time with ^18^F-FDG on LE was investigated. The prepared RBC suspension (100 μL, 70% hematocrit) was incubated for 60 min at 37 °C to reduce intracellular glucose concentration before labeling. The RBC suspension was mixed with ^18^F-FDG solution (11.1 ± 0.7 MBq, 14.3 ± 3.2 μL, *n* = 3). The labeling times with ^18^F-FDG at 37 °C were varied from 0 to 60 min at an interval of 5 min. Washing, radioactivity measurement, and LE calculation were performed following the same procedure as described above. Based on the results of these analyses, the optimal conditions for labeling RBCs with ^18^F-FDG were determined and then further experiments were performed.

### Post-labeling stability of ^18^F-FDG-labeled RBCs

The post-labeling stability was assessed by calculating a release fraction of radioactivity from ^18^F-FDG-labeled RBCs. The washed RBC suspension (1.5 ml, 80% hematocrit, *n* = 4) was incubated for 60 min at 37 °C to reduce intracellular glucose concentration before labeling. The RBC suspension was mixed with ^18^F-FDG (more than 100 MBq) and incubated for 30 min at 37 °C for labeling. To eliminate extracellular ^18^F-FDG, the labeled RBCs were washed three times with HBSS at 4 °C. The radioactivity of ^18^F-FDG-labeled RBCs was measured with the dose calibrator. The labeled RBC suspension was diluted with the plasma separated soon after blood sampling, and its hematocrit was adjusted to 40%. The labeled RBC suspension (200 μL, 40% hematocrit) was incubated for 0–180 min at 37 and 0 °C. After each incubation time, the labeled RBC suspension was cooled and uniformly resuspended. The whole RBC suspension (10 μL) was sampled for radioactivity measurement. The remnant RBC suspension was centrifuged at 1000*g* for 2 min, and the supernatant (10 μL) was also sampled. The whole RBC suspension and the supernatant were simultaneously measured with a well-type scintillation counter (ARC-380CL, Aloka, Japan). The release fraction was calculated according to the formula,

$$ \mathrm{Release}\ \mathrm{fraction}\ \left(\%\right)=\frac{\mathrm{Radioactivity}\ \mathrm{of}\ \mathrm{supernatant} \times \left(1-\frac{\mathrm{hematocrit}}{100}\right)}{\mathrm{Radioactivity}\ \mathrm{of}\ \mathrm{whole}\ \mathrm{RBC}\ \mathrm{suspension}} \times 100 $$

, where hematocrit was 40%.

In addition, extracellular glucose concentration of the supernatant during the 180-min incubation was measured with a glucometer to check the uptake ratio of glucose by RBCs at 37 and 0 °C.

### Thin-layer chromatography

To estimate the chemical form of ^18^F released from ^18^F-FDG-labeled RBCs, thin-layer chromatography (TLC) was performed. Fluorine-18-FDG-labeled RBC suspension diluted with plasma (40% hematocrit, 200 μL) was prepared following the same procedure used for the stability study described above. After a 60-min incubation of the ^18^F-FDG-labeled RBC suspension at 37 °C, it was centrifuged at 1000*g* for 2 min at 4 °C. The supernatant was spotted on to silica gel TLC strips (4 × 8 cm, Merck, Darmstadt, FRG). The packed RBCs were washed three times with HBSS at 4 °C to eliminate extracellular radioactivity, and the labeled RBCs were completely lysed with distilled water. The lysate was spotted onto another TLC strip. Original ^18^F-FDG solution was spotted as an authentic sample. Acetonitrile/water (*v*/*v* = 95:5) was used as eluent. Chromatograms were measured using a Typhoon FLA 9000 imager (GE Healthcare Life Sciences). Fluorine-18-FDG and ^18^F-FDG-6-phosphate were identified by their Rf values (0.37 and 0, respectively) [23].

### Preparation of labeled RBCs for PET imaging

After a 3-h fasting period, the rats were anesthetized with isoflurane. The blood (400 μL) was slowly withdrawn from the cervical vein using a heparinized syringe with a 27-gauge needle. After blood sampling, the rats were recovered from anesthesia and replaced in their cages without food. The blood was centrifuged at 500*g* for 5 min at 4 °C, and the buffy coat was removed. The packed RBC suspension (100 μL, 80% hematocrit) was washed three times with PBS. After a 60-min incubation in glucose-free PBS at 37 °C for reducing intracellular glucose concentration, the RBCs was mixed with ^18^F-FDG solution (20.0 ± 8.4 MBq) and incubated for 30 min at 37 °C for labeling. After labeling, ^18^F-FDG-labeled RBCs were washed three times with HBSS at 4 °C to eliminate extracellular ^18^F-FDG. The LE was calculated with decay correction. The packed labeled RBC suspension (100 μL) was diluted with HBSS, and the total volume was adjusted to 400 μL (20% hematocrit). The obtained labeled RBC suspension was cooled and preserved at 0 °C until injection. In addition, the labeled RBCs were observed with a microscope to evaluate their morphological abnormality.

### PET imaging of normal rats

The rats (169 ± 18*g*, *n* = 5) were anesthetized again and maintained with isoflurane throughout the imaging procedure. Imaging was performed using a small-animal PET system (ClairvivoPET; Shimadzu Corporation, Japan) [24]. The rats were placed in a supine position on a fixation plate in the PET scanner. A transmission scan with an external ^137^Cs point source (22 MBq) was performed for attenuation correction. Autologous ^18^F-FDG-labeled RBC suspension (10.5 ± 3.7 MBq, 400 μL, 20% hematocrit, rewarmed at room temperature) was injected via the lateral tail vein. Image acquisition was simultaneously started, and list-mode data were acquired for 120 min. The rectal temperature was kept at 36–38 °C by heating. The list-mode data were reconstructed into a dynamic sequence (27 frames, 3 × 10, 5 × 30, 2 × 60, 11 × 300, and 6 × 600 s) using 3D-DRAMA (dynamic row-action maximization-likelihood algorithm) with decay correction. The matrix size was 128 × 128 × 213 pixels.

### Intraabdominal bleeding models

We imaged intraabdominal bleeding models of rats as a potential use of ^18^F-FDG-labeled RBC PET. Before PET imaging, glycerin enema (1 mL) was transanally injected into the colon for defecation. After a transmission scan, autologous ^18^F-FDG-labeled RBC suspension (10 MBq, 400 μL) was intravenously injected, and image acquisition was simultaneously started. List-mode data were acquired for 40 min. In a steady-state of the BPI (20–25 min after injection), a transanal puncture of the colonic wall was manually given with an 18-gauge intravenous needle to induce hemorrhage into the abdominal cavity. In the present study, two kinds of bleeding models were imaged: persistent and temporary bleeding models. To model a persistent bleeding rat (171 g, *n* = 1), heparin (1000 IU/kg body weight) was injected via the tail vein just before injection of ^18^F-FDG-labeled RBCs, whereas no heparin was used for a temporary bleeding model (170 g, *n* = 1). After PET imaging, CT was performed using a micro CT system (R-mCT; Rigaku, Japan) to localize the extravascular blood inside the abdominal cavity. CT parameters were as follows: X-ray source, 90 kV/100 μA; rotation, 360°; exposure time, 17 s; and voxel size, 133 × 133 × 282 μm.

### Image analysis

The reconstructed images were analyzed using PMOD version 3.80 (PMOD Technologies Ltd, Switzerland). In the images of normal rats, volumes of interest (VOIs) were placed in each organ, and the radioactivity was expressed as the percentage of injected dose per gram (%ID/g). Time-activity curves (TACs) for the atrium, lung, liver, brain, spleen, kidney, and urinary bladder were obtained.

In order to measure the radioactivity of bleeding in the intraabdominal bleeding models, image subtraction was performed between the post-bleeding images (38–40 min after injection) and the pre-bleeding images (10–20 min after injection) with an algebra tool of PMOD. Negative values of the pixels in the subtracted images were all replaced to zero value. Then, the total radioactivity of bleeding during PET imaging was measured as the percentage of injected dose (%ID) after excluding radioactivity of the urinary bladder.

### Statistical analysis

Quantitative data were expressed as mean ± SD. Spearman’s rank correlation coefficient was used to assess the quality of linear correlations using the SPSS software package (version 22.0, SPSS Inc., IBM, Chicago, USA). A *P* value of less than 0.05 was considered statistically significant.

## Results

### Labeling efficiency

**Fig. 1:**
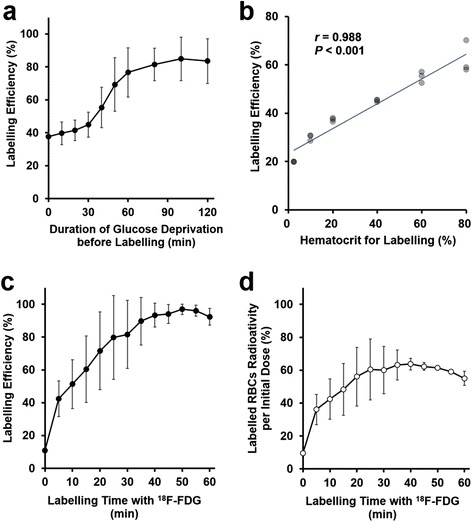
Relationship between labeling efficiency and its influence factors. **a** Effect of the preparation of glucose deprivation before labeling on labeling efficiency (labeling time, 30 min; *n* = 4). **b** Relationship between hematocrit for labeling and labeling efficiency. A positive linear correlation was found (*some dots overlap*). **c** Relationship between labeling time with ^18^F-FDG and labeling efficiency (glucose deprivation time, 60 min; *n* = 3). **d** Relationship between labeling time with ^18^F-FDG and the percentage of the actual radioactivity of ^18^F-FDG-labeled RBCs per the initial dose (the same data as Fig. 1c expressed without decay correction)

The blood glucose concentration soon after blood sampling was 232 ± 30 mg/dL. In Fig. 1, LE under various circumstances is summarized. The LE increased with the incubation time in PBS before labeling and was 37.6% in 0 min and 76.7% in 60 min (Fig. 1a). The higher the hematocrit for labeling is, the higher the LE is (Fig. 1b). At this time, there were technical difficulties accurately measuring LE when hematocrit was over 80% because of the high viscosity and the presence of dried-up RBCs around fluid surface during a long incubation. Even though LE continued to rise over labeling time (Fig. 1c), the loss of radioactivity exceeded the increase in LE after 30–35 min and the net radioactivity began to decrease after this time (Fig. 1d). There was a positive correlation between ^18^F-FDG-added and ^18^F-FDG-labeled RBCs (Additional file 1: Figure S1). Based on these results, we determined that the optimal labeling conditions were as follows: glucose depriving time was 60 min, hematocrit for labeling was 70%, and labeling (incubation) time was 30 min.

### Post-labeling stability of ^18^F-FDG-labeled RBCs

**Fig. 2:**
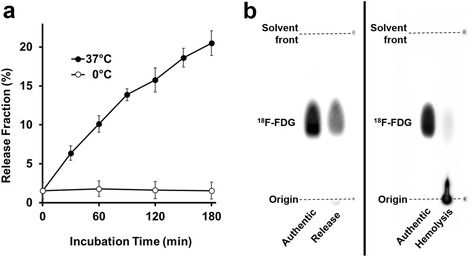
Post-labeling stability analysis. **a** Release fraction of the radioactivity from ^18^F-FDG-labeled RBCs during a 180-min incubation in plasma at 37 or at 0 °C (*n* = 4). **b** Thin-layer chromatography of extra- (*left*, release) and intracellular fluid (*right*, hemolysis) after a 60-min incubation of ^18^F-FDG-labeled RBCs in plasma. Obvious difference between them was seen

In Table 1, LE of ^18^F-FDG-labeled RBCs by means of the optimized labeling procedure is shown. The obtained radioactivity of ^18^F-FDG-labeled RBC suspension (1.5 mL) was 89 ± 26 MBq, and the LE was 69.3 ± 13.3%. Figure 2 summarizes the post-labeling stability of ^18^F-FDG-labeled RBCs. The release fraction of a chemical form of ^18^F increased constantly for 3 h at 37 °C; no release was seen at 0 °C (Fig. 2a). The release fractions at 37 °C in 60, 120, and 180 min incubation were 10.1, 15.8, and 20.5%, respectively, which were larger than we expected. TLC of extra- and intracellular fluid at 60 min of incubation showed the ^18^F-FDG ratio of extracellular fluid (release) was 92.6%, while that of intracellular fluid (hemolysis) was 4.7% (Fig. 2b). Extracellular glucose concentration gradually decreased at 37 °C but did not change at 0 °C (Additional file 1: Figure S2).

**Table 1:** Labeling efficiency of ^18^F-FDG-labeled RBCs by the optimized labeling procedure

Prepared RBC suspension	^18^F-FDG used for labeling	^18^F-FDG-labeled RBCs	Labeling efficiency^a^
1.5 ml (80% hematocrit)	166 ± 29 MBq	89 ± 26 MBq	69.3 ± 13.3%

**Fig. 3:**
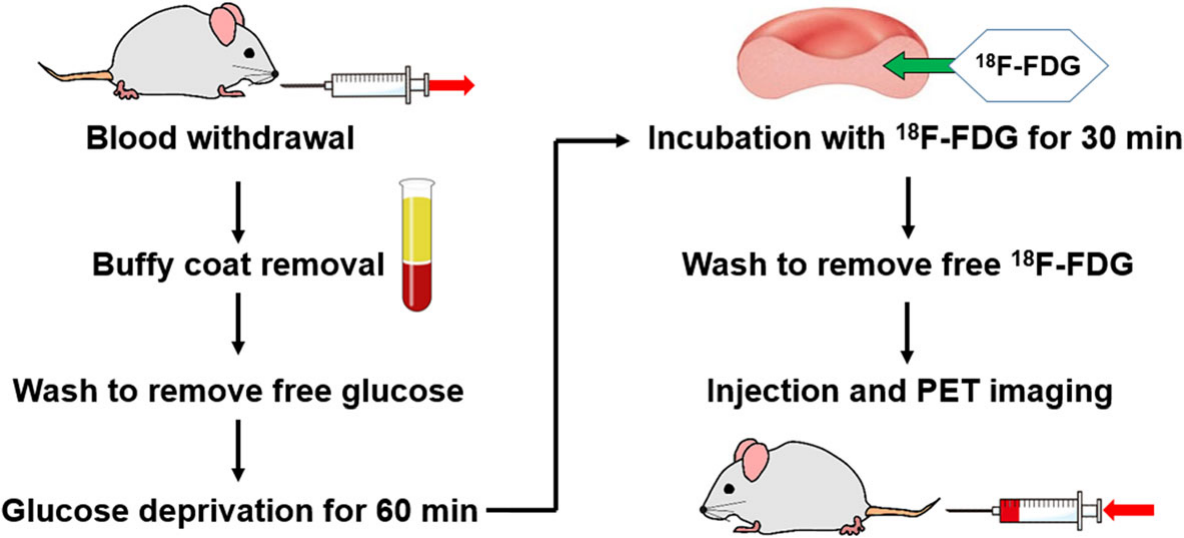
An optimal labeling procedure of ^18^F-FDG-labeled RBCs for in vivo imaging of rats

Data are mean ± SD (*n* = 4). The optimized labeling procedure is described in Fig. 3

^a^Decay corrected

### Labeling for in vivo imaging

Labeling RBCs with ^18^F-FDG for in vivo imaging was performed with the optimized procedure as shown in Fig. 3. The average time from blood sampling to intravenous injection of ^18^F-FDG-labeled RBC suspension was approximately 125 min. In microscopic images, ^18^F-FDG-labeled RBCs appeared normal and no aggregation of RBCs nor RBCs with abnormal shapes (e.g., spherocytes, acanthocytes, or schistocytes) were seen (Additional file 1: Figure S3). The radioactivity of the obtained ^18^F-FDG-labeled RBC suspension (400 μL, 20% hematocrit) were 11.2 ± 3.9 MBq, and the LE was 71.7 ± 10.2% (*n* = 4).

### PET imaging of normal rats

**Fig. 4:**
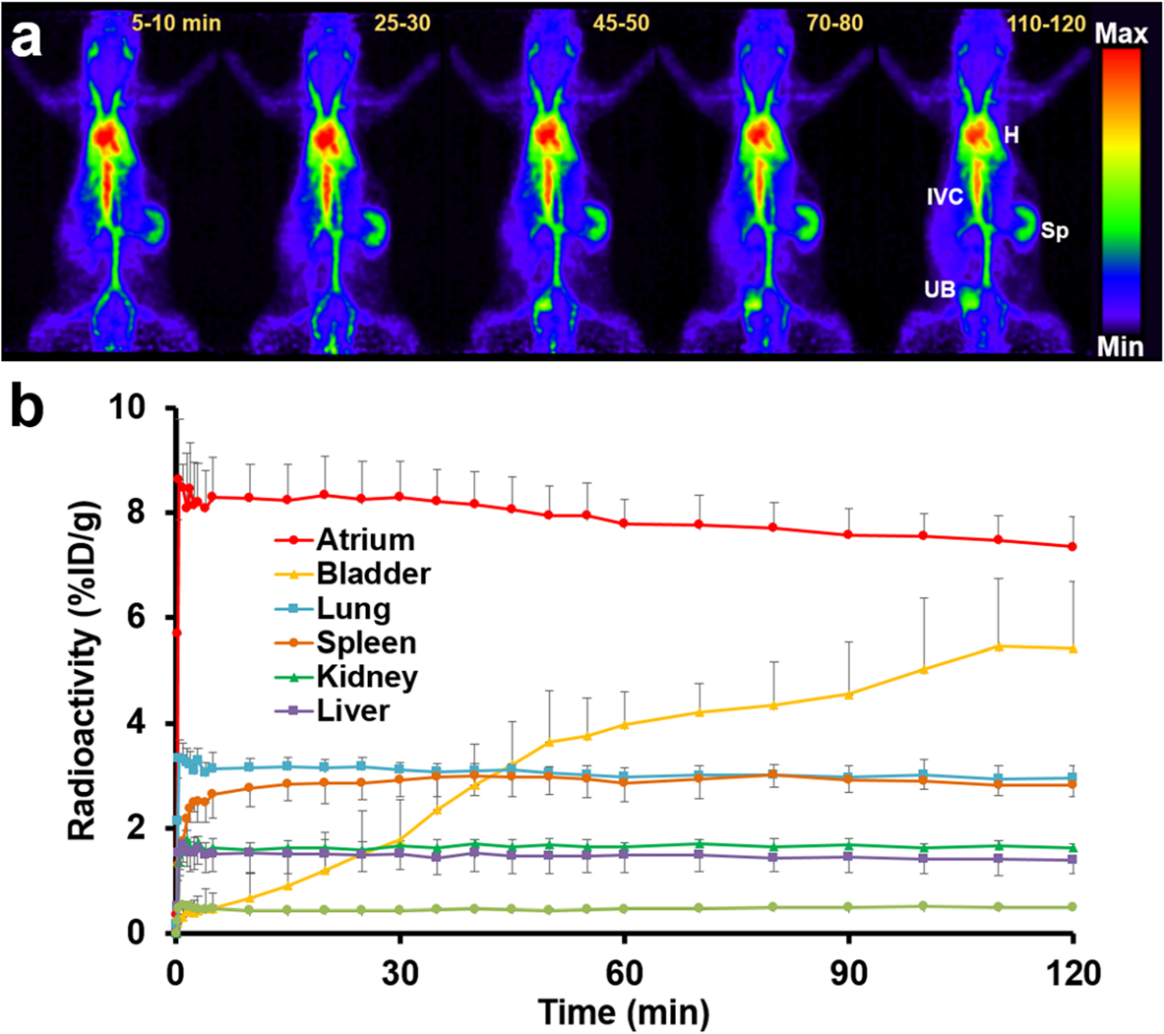
Whole body data of normal rats in ^18^F-FDG-labeled RBC PET. **a** Representative serial maximum-intensity-projection images after intravenous injection for 120 min of ^18^F-FDG-labeled RBCs. Cardiac cavities and vascular system were clearly visualized. *H* heart, *IVC* inferior vena cava, *Sp* spleen, *UB* urinary bladder. **b** Time-activity curves of the organs during PET imaging for 120 min (*n* = 5)

The maximum-intensity-projection images revealed that the cardiac space and the vessels of whole bodies showed a strong accumulation of ^18^F-FDG-labeled RBCs (Fig. 4a). TACs of the organs showed that the radioactivity of the most organs was relatively constant up to 120 min (Fig. 4b), suggesting that our procedure was appropriate for BPI. The intraatrial radioactivity of 60 min decreased only 5.6% compared to that of 5–10 min. Meanwhile, slight urinary excretion was seen. The splenic radioactivity was higher than the hepatic radioactivity and lower than the pulmonary radioactivity during 30 min after injection.

### Intraabdominal bleeding models

**Fig. 5:**
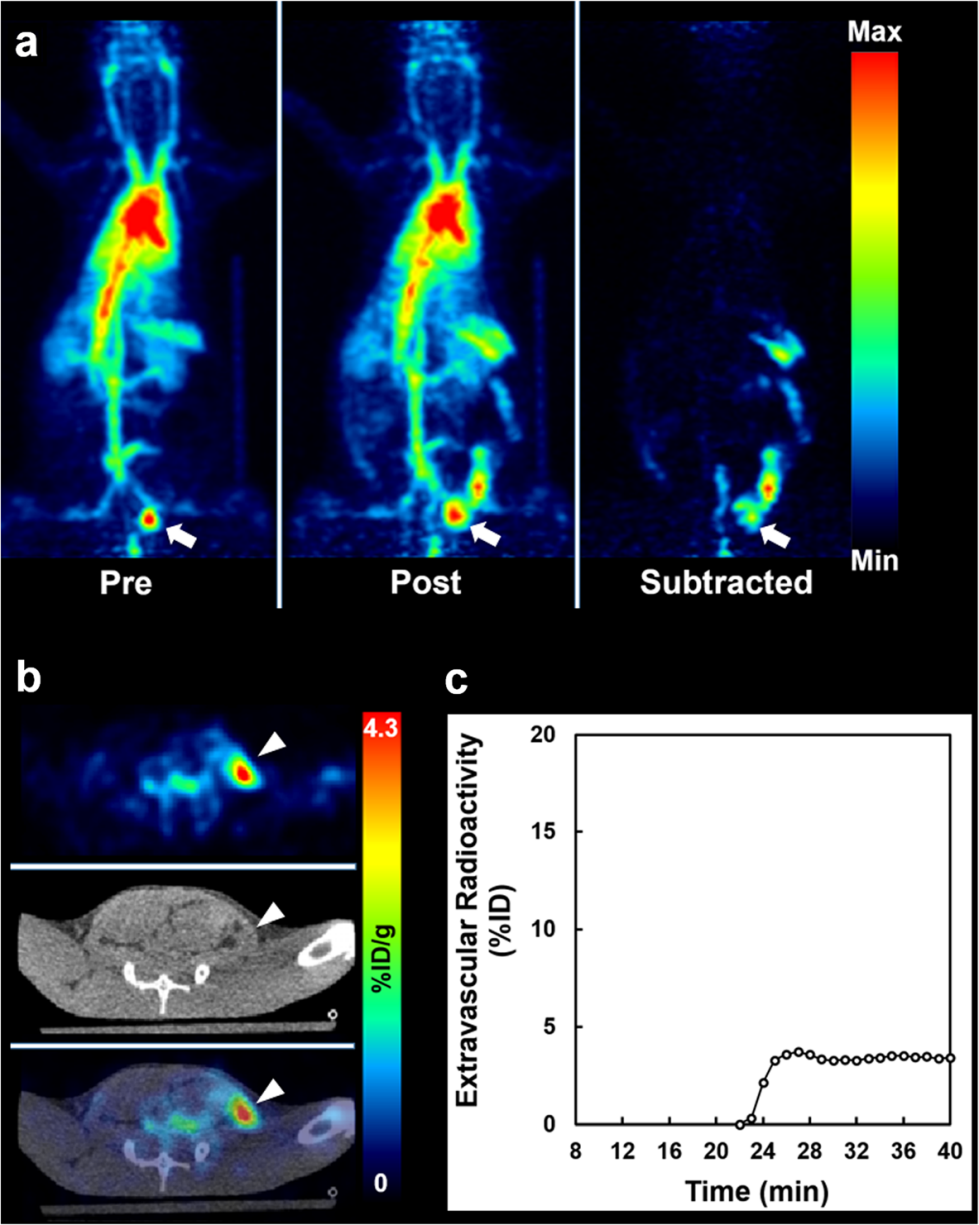
^18^F-FDG-labeled RBC PET in a temporary intraabdominal bleeding model. **a** Maximum-intensity-projection images in pre- (*left*), post- (*middle*) bleeding phase, and the subtracted (right) image between them. The radioactivity in the urinary bladder is seen in the images (*arrows*). **b** PET, CT, and fused PET/CT axial images at lower level of the abdomen. Intraabdominal bleeding is identified as high radioactivity in the extravascular space in the fused image (*arrowheads*). **c** Time-activity curve of extravascular radioactivity in the subtracted image. The radioactivity appeared soon after the manual puncture of the colonic wall (22 min), and then it reached a plateau about 5 min later

**Fig. 6:**
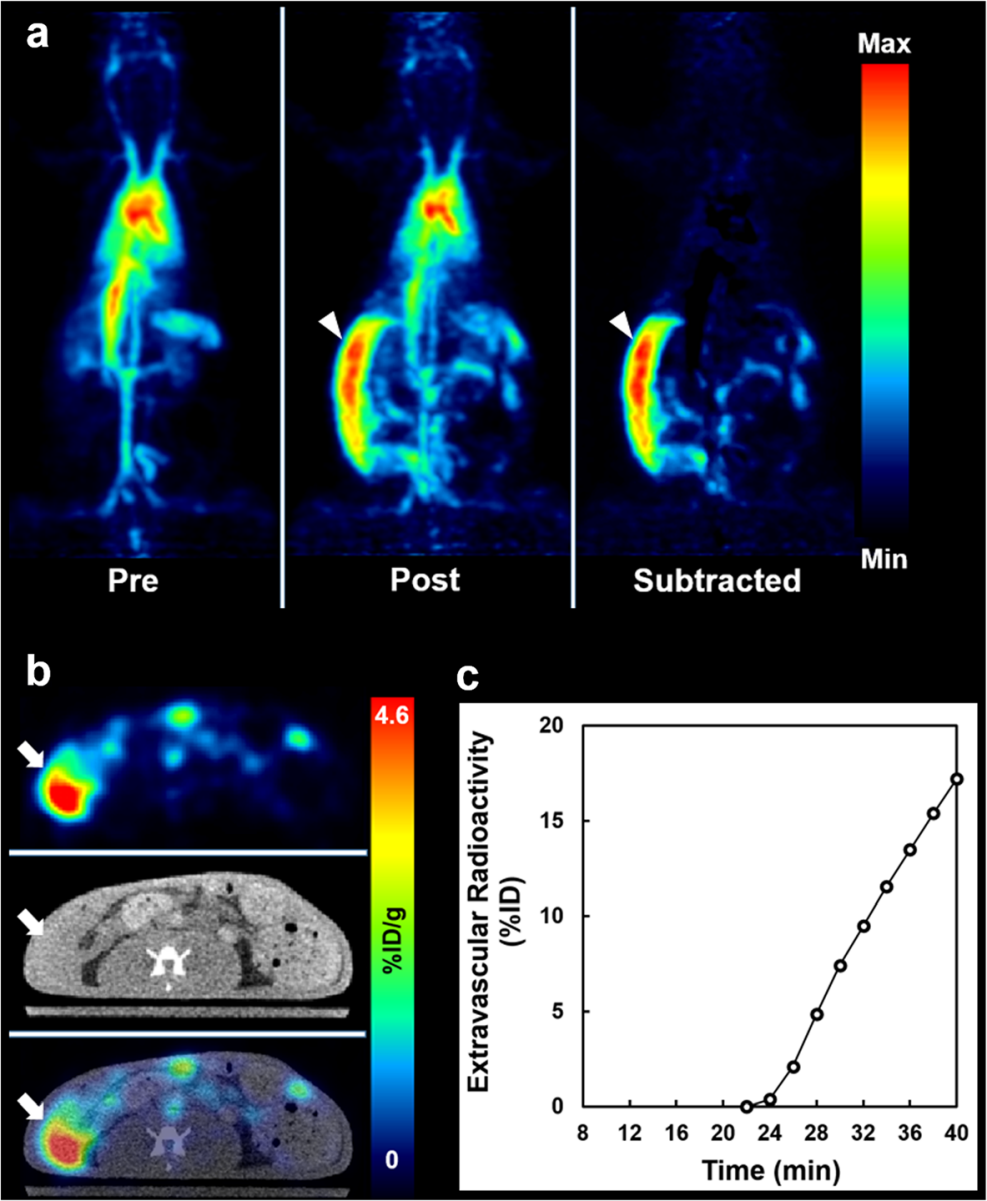
^18^F-FDG-labeled RBC PET in a persistent intraabdominal bleeding model. **a** Maximum-intensity-projection images in pre- (*left*) and post- (*middle*) bleeding phase and the subtracted (*right*) image between them. The subtracted image shows massive bleeding in the right side of the abdomen (*arrowheads*). **b** PET, CT, and fused PET/CT axial images at the middle level of the abdomen. CT shows an area of fluid collection, and the fused image shows high radioactivity in the corresponding area (*arrows*). **c** Time-activity curve of extravascular radioactivity in the subtracted image. The radioactivity was continuously increased after the manual puncture of the colonic wall (22 min)

Dynamic PET images revealed the appearance of high accumulation sites in the abdomen soon after the puncture of the colon wall, suggesting the extravasation of the tracer due to bleeding (Figs. 5a and 6a). Extravascular blood was not identified in the CT image (Figs. 5b and 6b). The TAC of the extravascular blood in the temporary bleeding model (without heparin) rose soon after puncture of the colon wall and reached a plateau in 4 min, while the TAC in the persistent model (with heparin) rose continuously (Figs. 5c and 6c).

## Discussion

To our knowledge, this is the first study demonstrating the feasibility of labeling RBCs with ^18^F-FDG. In order to apply ^18^F-FDG-labeled RBC PET to human study, optimization or standardization of labeling procedure is crucial in terms of the accuracy of ^18^F-FDG-labeled RBC PET for BPI. Therefore, we sought the optimal labeling procedure to achieve the highest LE and stability by measuring LE in different conditions. In the optimized labeling protocol, ^18^F-FDG-labeled RBC PET revealed high retention of the tracer in the cardiovascular system for at least 120 min in normal rats. In the intraabdominal bleeding models, ^18^F-FDG-labeled RBC PET visualized the extravascular blood more clearly than CT and revealed the different patterns of the extravascular radioactivity between the temporary and persistent bleeding models.

We considered that high LE could be achieved by depriving glucose from RBCs. Glucose deprivation reduces intracellular glucose concentration. In addition, a glucose concentration gradient across the membrane is the driving force to transport glucose via glucose transporter-1 on the plasma membrane of RBCs [25]. Taken together, this pre-labeling preparation can boost the uptake of glucose or ^18^F-FDG into RBCs. Indeed, our results showed that glucose deprivation for up to 60 min significantly improved the LE (Fig. 1a). Considering the balance between the LE and the potential cell damage due to prolonged glucose deprivation, the duration of glucose deprivation was set at 60 min in subsequent studies. Higher hematocrit was suitable for achieving higher LE (Fig. 1b). However, it was practically difficult to uniformly mix high-hematocrit RBC suspension (over 80% hematocrit, 100 or 200 μL) with ^18^F-FDG solution (10–20 μL) due to high viscosity. In addition, some RBCs in high-hematocrit RBC suspension dried up around fluid surface during long incubation. Therefore, hematocrit was set at 70% in other labeling studies. LE steadily increased during the incubation with ^18^F-FDG, reaching a plateau in 50 min (Fig. 1c). Consequently, the total amount of radioactivity in the RBCs showed a peak from 30 to 40 min due to beta decay of ^18^F (Fig. 1d). As a result, the optimal incubation (labeling) time with ^18^F-FDG was considered to be 30 min.

In the stability studies in vitro, ^18^F-FDG-labeled RBCs gradually released ^18^F-FDG at 37 °C for at least 3 h (Fig. 2). Since the released ^18^F-FDG would be excreted in urine or taken up by extravascular tissues after injection, BPI with ^18^F-FDG-labeled RBCs should be performed as early as possible after equilibrium in the blood is achieved. While the retention rate of ^18^F-FDG in the in vitro assessment was 89.9% at 60 min (Fig. 2a), that of the stability studies in the in vivo assessment was 94.4% at 60 min (Fig. 4b). Therefore, we concluded that ^18^F-FDG-labeled RBCs were relatively stable in vivo and were suitable for BPI.

We found that keeping the labeled RBCs at 0 °C prevented the ^18^F-FDG release. Thus, the preparation after the incubation with ^18^F-FDG should be performed at 0° in case of a long time interval between the preparation and injection. Actually, we performed pre-injection cooling and rewarming just before injection for in vivo studies.

Based on the results of TLC, the radioactive substances inside ^18^F-FDG-labeled RBCs during the incubation in plasma were almost all non-FDG (Fig. 2b). Radioactivity in the intracellular fluid was likely to represent ^18^F-FDG-6-phosphate because of the phosphorylation by hexokinase in RBCs. In addition, the fact that RBCs have no radioactivity of glucose-6-phosphatase [26] would account for the small amount (<5% in 60 min after incubation) of ^18^F-FDG. However, the exact mechanism for increasing extracellular ^18^F-FDG (i.e., the labeled RBCs continued to release ^18^F-FDG) over 180 min was unknown. There might be a glucose-6-phosphatase-independent mechanism as suggested in some tumor cells [27].

An optimal labeling procedure for the in vivo imaging of rats is shown in Fig. 3. After injecting ^18^F-FDG-labeled RBCs intravenously, PET images exhibited marked contrast for the blood pool (Fig. 4a). TACs showed that the radioactivity of most organs was nearly unchanged after equilibrium was achieved (Fig. 4b). Since we used in vitro labeling technique, denaturation of the labeled RBCs may be suspected. However, whether ^18^F-FDG-labeled RBCs were denaturated was unknown because splenic radioactivity was not as high as typically seen in denaturation of the labeled RBCs. Splenic radioactivity depends not only on denaturation of RBCs but also on splenic sequestration of RBCs [28]. Furthermore, splenic sequestration is influenced by adrenergic agonists [28, 29] and isoflurane anesthesia [29]. Thus, the elevation of splenic radioactivity in the present study did not necessarily mean the occurrence of RBC denaturation. Splenic radioactivity of our normal rats during 30 min after injection (Fig. 4b) was almost the same as that of the previous study using ^11^C-CO-labeled RBCs in rats [13], in which splenic radioactivity was higher than hepatic radioactivity and lower than pulmonary radioactivity during 30 min after agent administration. Since the labeling procedure of ^11^C-CO-labeled RBCs is only an inhalation of ^11^C-CO gas, splenic radioactivity of ^11^C-CO-labeled RBCs would not be affected by RBC denaturation. Therefore, it is considered that the ^18^F-FDG-labeled RBCs produced in our procedure would be as stable as ^11^C-CO-labeled RBCs in rats.

In the intraabdominal bleeding models, the extravascular blood was three-dimensionally identified on the PET images and the dynamic change of extravascular radioactivity was observed (Figs. 5 and 6). Although this was challenging, we tried to measure the change of extravascular radioactivity in the two different intraabdominal bleeding models (Figs. 5c and 6c) because the bleeding rate as well as the bleeding point or volume can influence treatment strategy for intraabdominal bleeding in human practice [1]. The differences in aggressiveness or time course of intraabdominal bleeding could be observed with ^18^F-FDG-labeled RBC PET, although the quantitative accuracy was not confirmed in the present study. Future evaluation is needed to support this potential way to use ^18^F-FDG-labeled RBC PET.

Labeling RBCs with ^18^F-FDG should be of greater value than the other PET tracers because of the overwhelming availability and accessibility of ^18^F-FDG. However, there are some limitations to our method. First, in vitro labeling is troublesome due to the necessity of blood sampling and labeling procedure, the requirement for a clean space, and the radiation exposure for operators. We may be able to simplify the labeling procedures by reducing the number of washes or by shortening the duration of glucose deprivation. In the present study, we introduced the surest way to obtain BPI using ^18^F-FDG. Further evaluation regarding simplification of the labeling technique is needed. Second, ^18^F-FDG-labeled RBC PET has many issues towards clinical applications in contrast to ^99m^Tc-labeled RBC scintigraphy. Although RBCs can be labeled with ^99m^Tc with the in vivo method [30], it seems to be impossible to label RBCs with ^18^F-FDG in vivo. Given the short half-life of ^18^F, it is unlikely that any institution would have a dose available for an emergency GI bleeding study at midnight. Even at daytime, it is not easy to keep ^18^F-FDG available for unpredictable emergency procedures. Thus, ^18^F-FDG-labeled RBC PET is challenging for clinical translation at present.

## Conclusions

We presented an optimal labeling procedure of ^18^F-FDG-labeled RBCs for BPI. Among the factors that influenced LE, we found that glucose deprivation dramatically increased LE. Although a slight fraction of ^18^F-FDG was released from ^18^F-FDG-labeled RBCs, ^18^F-FDG-labeled RBC PET images revealed that the cardiovascular system of normal rats was clearly and stably visualized for several hours. In the intraabdominal bleeding models, the extravascular blood was clearly visualized on PET and the dynamic change of extravascular radioactivity was evaluated. There is the need for further research to determine the potential of ^18^F-FDG-labeled RBCs as a clinical agent for BPI in humans.

## Additional file

Additional file 1: Figure S1. Relationship between the radioactivity of added ^18^F-FDG and the actual radioactivity of ^18^F-FDG-labeled RBCs. Positive linear correlation between them was found (some dots overlap). **Figure S2.** Extracellular glucose concentration during a 180-min incubation of ^18^F-FDG-labeled RBCs in non-radioactive plasma at 37 °C and at 0 °C (*n* = 4). **Figure S3.** Representative microscopic appearance of ^18^F-FDG-labeled RBCs sampled just before injection. Abnormal RBCs were not identified. (DOCX 330 kb)

## Abbreviations

^18^F-FDG: ^18^F-2-deoxy-2-fluoro-D-glucose
^18^F-SFB: ^18^F-N-succinimidyl 4-^18^F-fluorobenzoate
BPI: Blood-pool imaging
CO: Carbon monoxide
CT: Computed tomography
IVC: Inferior vena cava
LE: Labeling efficiency
PBS: Phosphate-buffered saline
PET: Positron emission tomography
RBC: Red blood cell
SPECT: Single-photon emission computed tomography
TAC: Time-activity curve
TLC: Thin-layer chromatography
VOI: Volume of interest
WBC: White blood cell
