# Supplementary material for: 18F-FDG-labeled red blood cell PET for blood-pool imaging: preclinical evaluation in rats
Source: EJNMMI Res. 2017 Feb 27;7:19. doi: 10.1186/s13550-017-0266-3 (PMC5328895; doi:10.1186/s13550-017-0266-3)
Supplement: Additional file 1: Figure S1. — Relationship between the radioactivity of added 18F-FDG and the actual radioactivity of 18F-FDG-labeled RBCs. Positive linear correlation between them was found (some dots overlap). Figure S2. Extracellular glucose concentration during a 180-min incubation of 18F-FDG-labeled RBCs in non-radioactive plasma at 37 °C and at 0 °C (n = 4). Figure S3. Representative microscopic appearance of 18F-FDG-labeled RBCs sampled just before injection. Abnormal RBCs were not identified. (DOCX 330 kb) [file 13550_2017_266_MOESM1_ESM.docx]

**Additional file 1**


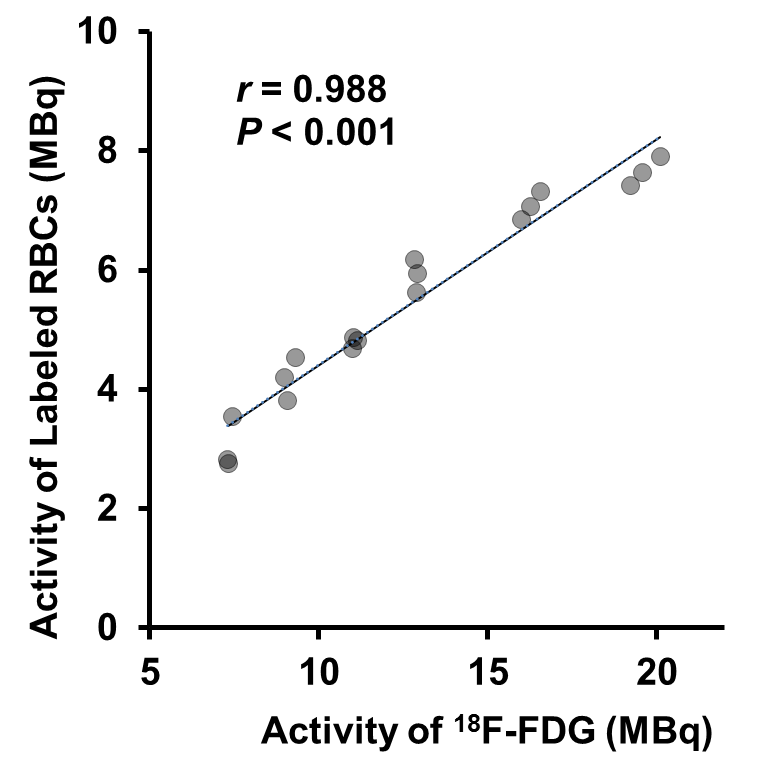


**Figure S1**

Relationship between the radioactivity of added ^18^F-FDG and the actual radioactivity of ^18^F-FDG-labelled RBCs. Positive linear correlation between them was found (some dots overlap).


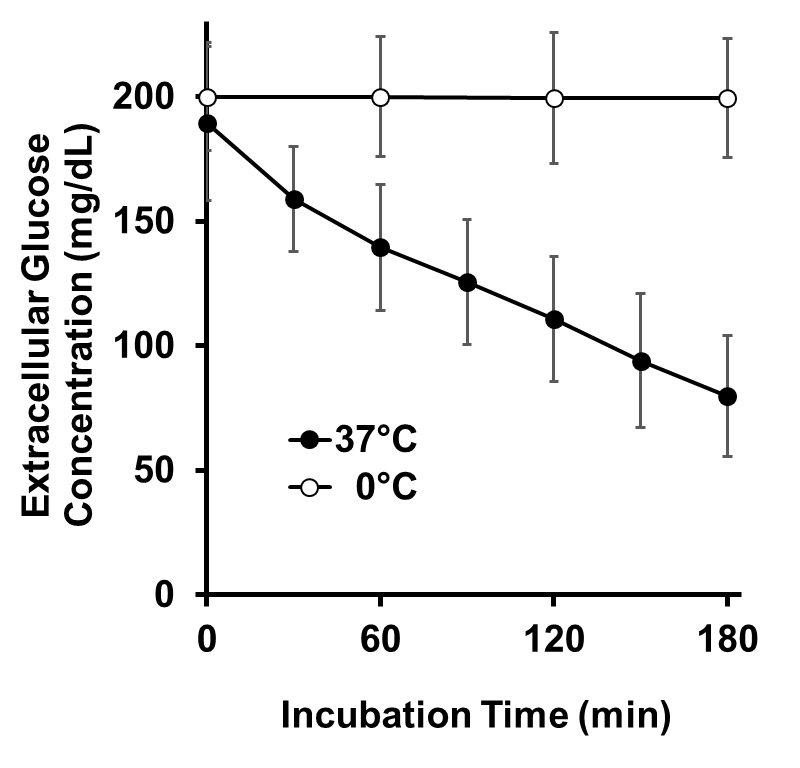


**Figure** **S2**

Extracellular glucose concentration during a 180-min incubation of ^18^F-FDG-labelled RBCs in non-radioactive plasma at 37°C and at 0°C (*n* = 4).


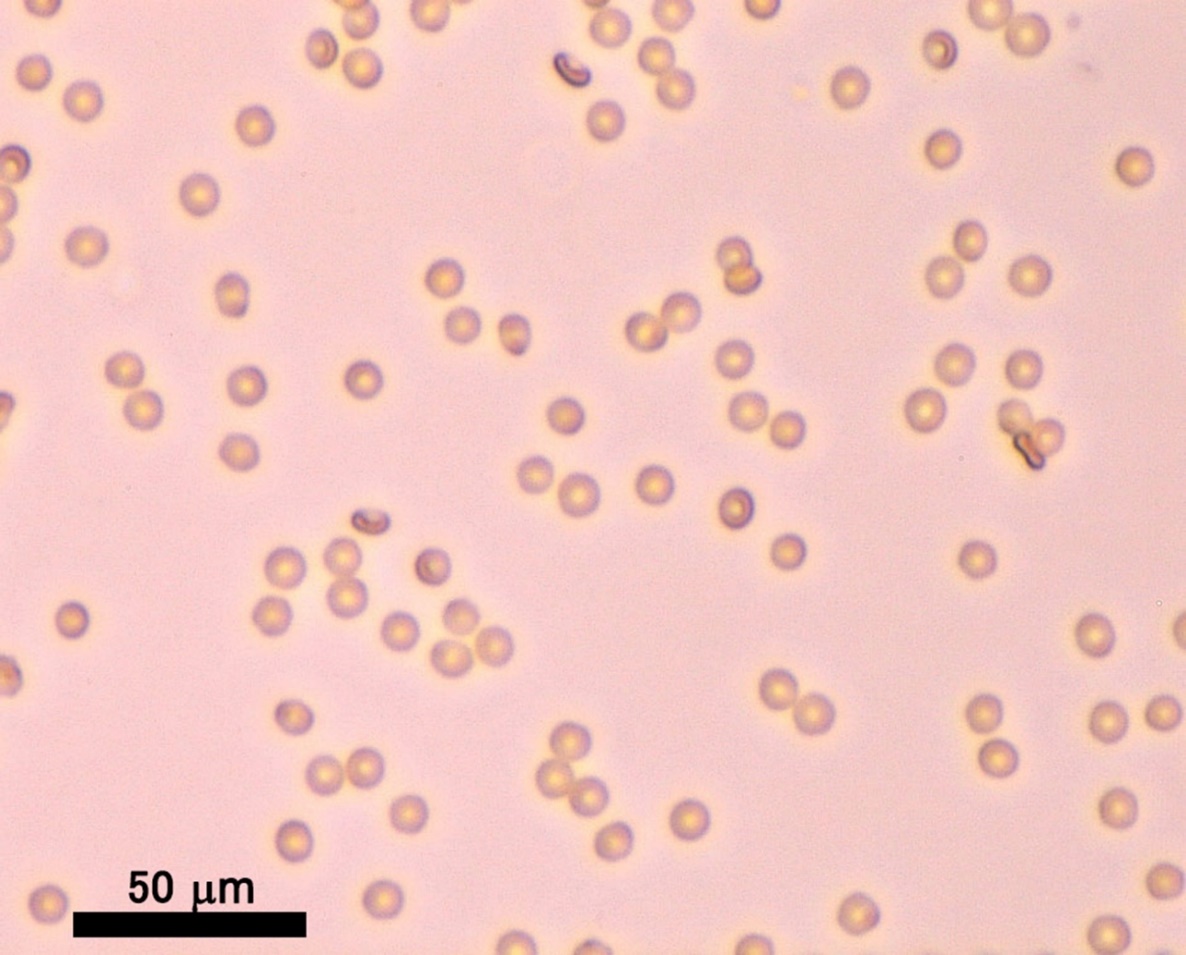


**Figure S3**

Representative microscopic appearance of ^18^F-FDG-labelled RBCs sampled just before injection. Abnormal RBCs were not identified.
